# Inhibition of Connexin 26/43 and Extracellular-Regulated Kinase Protein Plays a Critical Role in Melatonin Facilitated Gap Junctional Intercellular Communication in Hydrogen Peroxide-Treated HaCaT Keratinocyte Cells

**DOI:** 10.1155/2012/589365

**Published:** 2012-11-08

**Authors:** Hyo-Jung Lee, Hyo-Jeong Lee, Eun Jung Sohn, Eun-Ok Lee, Jin-Hyoung Kim, Min-Ho Lee, Sung-Hoon Kim

**Affiliations:** ^1^College of Oriental Medicine, Kyung Hee University, 1 Hoegi-dong, Dongdaemun-gu, Seoul 131-701, Republic of Korea; ^2^College of Life Sciences and Biotechnology, Kyung Hee University, Yongin 446-701, Republic of Korea

## Abstract

Though melatonin was known to regulate gap junctional intercellular communication (GJIC) in chick astrocytes and mouse hepatocytes, the underlying mechanism by melatonin was not elucidated in hydrogen peroxide- (H_2_O_2_-) treated HaCaT keratinocyte cells until now. In the current study, though melatonin at 2 mM and hydrogen peroxide (H_2_O_2_) at 300 **μ**M showed weak cytotoxicity in HaCaT keratinocyte cells, melatonin significantly suppressed the formation of reactive oxygen species (ROS) in H_2_O_2_-treated HaCaT cells compared to untreated controls. Also, the scrape-loading dye-transfer assay revealed that melatonin enhances the intercellular communication by introducing Lucifer Yellow into H_2_O_2_-treated cells. Furthermore, melatonin significantly enhanced the expression of connexin 26 (Cx26) and connexin 43 (Cx43) at mRNA and protein levels, but not that of connexin 30 (Cx30) in H_2_O_2_-treated HaCaT cells. Of note, melatonin attenuated the phosphorylation of extracellular signal-regulated protein kinases (ERKs) more than p38 MAPK or JNK in H_2_O_2_-treated HaCaT cells. Conversely, ERK inhibitor PD98059 promoted the intercellular communication in H_2_O_2_-treated HaCaT cells. Furthermore, combined treatment of melatonin (200 **μ**M) and vitamin C (10 **μ**g/mL) significantly reduced ROS production in H_2_O_2_-treated HaCaT cells. Overall, these findings support the scientific evidences that melatonin facilitates gap junctional intercellular communication in H_2_O_2_-treated HaCaT keratinocyte cells via inhibition of connexin 26/43 and ERK as a potent chemopreventive agent.

## 1. Introduction

Gap junctional intercellular communication (GJIC) is an important biological mechanism to maintain homeostasis, growth, differentiation, and development of cells and tissues [1]. Gap junctions are made of two hemichannels, called connexons, and each in turn is composed of six molecules of the membrane-spanning connexin (Cx) protein [2, 3].

The gap junctions of human keratinocytes include primarily Cx43, which is abundantly expressed within interfollicular epidermis, and Cx26, which is codistributed with Cx43 in skin [4]. Several studies showed that the downregulation of Cxs and phosphorylation of Cxs are involved in the carcinogenesis of the skin [4, 5]. Cx43 is phosphorylated by several protein kinases, such as protein kinase C (PKC), casein kinase 1, and mitogen-activated protein kinase (MAPK) [3, 6–8]. Recent evidence suggests that the carcinogenicity of oxidative stress induced by H_2_O_2_ is attributable to the inhibition of GJIC [8–10].

Melatonin, an indoleamine (N-acetyl-5 methoxytryptamine), produced especially at night in the pineal gland [11, 12], has antioxidant [13, 14], anti-inflammatory [15, 16], antidepressant [17], and antitumor activities against various cancers [18–20]. Though melatonin was recently shown to regulate GJIC in chick astrocyte [21], mouse hepatocytes [22], and MCF-7 breast cancer cells [23, 24], the underlying molecular mechanism by melatonin via GJIC regulation in human keratinocyte HaCaT cells still remains unclear. Thus, in the present study, the molecular mechanism responsible for GJIC regulation by melatonin was examined in human keratinocyte HaCaT cells using the MTT assay, scrape-loading assay, RT-PCR, western blotting, and flow cytometric analysis for reactive oxygen species (ROS).

## 2. Materials and Methods

### 2.1. Chemicals and Reagents

Melatonin (molecular weight: 232), dimethylsulfoxide (DMSO), 3-(4,5-dimethylthiazol-2-yl)-2,5-diphenyltetrazolium bromide (MTT), protease inhibitor cocktail, Lucifer Yellow, Trizol reagent, MMLV, Taq polymerase, vitamin C, and 2,7-dichlorofluorescein diacetate (DCFDA) fluorescence dye were purchased from Sigma-Aldrich (St. Louis, MO, USA). Primers (Cx26, Cx30, and Cx43) were purchased from Cosmogenetech (Seoul, Republic of Korea). Dulbecco's Modified Eagle Medium (DMEM), fetal bovine serum (FBS), and antibiotic-antimycotic agent were obtained from Welgene (Daegu, Republic of Korea). Sodium dodecyl sulfate (SDS) was purchased from Amresco (Solon, OH, USA). RC DC protein assay kit was purchased from Bio-Rad (Hercules, CA, USA). Dimethylformamide was obtained from Merck KGaA (Darmstadt, Germany). Enhanced chemiluminescence (ECL) detection reagent was purchased from Amersham Pharmacia (Piscataway, NJ, USA). Phospho-JNK, JNK phospho-p38 MAPK, p38 MAPK, phospho-ERK, and ERK antibodies were obtained from Cell Signaling Technology (Danvers, MA, USA). Cx26, Cx30, Cx43, and phospho-Cx43 antibodies were purchased from Santa Cruz Biotechnology (Santa Cruz, CA, USA). *β*-actin was purchased from Sigma-Aldrich (St. Louis, MO, USA). Melatonin was dissolved in DMSO (2 M stock solution). In all experiments, DMSO concentration was kept below 0.2% (v/v) to remove the cytotoxic effect of solvent DMSO.

### 2.2. Cell Culture

Human keratinocyte HaCaT cells were purchased from American Type Culture Collection (Manassas, VA, USA) and maintained in DMEM supplemented with 10% FBS and penicillin/streptomycin.

### 2.3. Cytotoxicity Assay

The cytotoxicity of melatonin was measured by MTT colorimetric assay. HaCaT cells were seeded onto 96-well microplates at a density of 1 × 10^4^ cells per well and treated with various concentrations of melatonin for 24 h. MTT working solution (5 mg/mL in PBS) was added to each well and incubated at 37°C for 3 h. The optical density (OD) was then measured at 570 nm using a microplate reader (Sunrise, TECAN, Männedorf, Switzerland). Cell viability was calculated as a percentage of viable cells in melatonin or H_2_O_2_-treated group versus untreated control by the following equation: cell viability (%) = [OD (melatonin) − OD (blank)]/[OD(Control) − OD (Blank)] × 100.

### 2.4. Scrape-Loading Dye-Transfer Assay

GJIC of the cells was assessed by the scrape-loading dye-transfer (SLDT) technique described by EL-Fouly et al. [25] with some modifications. HaCaT cells (cell confluency; 80–90%) incubated in 35 mm dishes for 24 h were treated with H_2_O_2_ (300 *μ*M) or melatonin (1 or 2 mM), respectively. Following incubation, the cells were washed twice with 2 mL of PBS. Lucifer Yellow was added to the washed cells, and three scrapes were made with a surgical steel-bladed scalpel at low-light intensities. Three scrapes were performed to ensure that the scrape traversed a large group of confluent cells. After 3 min incubation, the cells were washed with 10 mL of PBS and then fixed with 2 mL of a 4% formalin solution. The distance traveled by the dye in a direction perpendicular to the scrape was observed with an inverted Axio Axiovert S 100 fluorescent microscope (Carl Zeiss).

### 2.5. Total RNA Isolation and RT-PCR Analysis

Total RNA was prepared by using Trizol reagent according to the manufacturer's instructions. Total RNA (1.0 *μ*g) was reverse transcribed using MMLV reverse transcriptase (Promega, Madison, WI, USA) by incubation at 25°C for 10 min, at 42°C for 60 min, and at 99°C for 5 min. The synthesized cDNA was amplified using TaKaRa Taq DNA polymerase (TaKaRa Biotechnology, Shiga, Japan) and the following specific primers: *Cx26* (sense 5′-TCTTTTCCAGAGCAAACCGC-3′; antisense 5′-CTGGGCAATGAGTTAAACTGG-3′), *Cx30* (sense 5′-GCAGCATCTTTTTCCGAATC-3′; antisense 5′-ATGCTCCTTTGTCAAGACGT-3′), *Cx43* (sense 5′-TACCATGCGACCAGTGGTGCGCT-3′, antisense 5′-GAATTCTGGTTATCATCGGGGAA-3′), and *GAPDH* (sense 5′-GTGGATATTGTTGCCATCA-3′, antisense 5′-ACTCATACAGCACCTCAG-3′). PCR conditions were 30 cycles of 96°C for 30 sec, 55°C for 30 sec, and 72°C for 30 sec, followed by 5 min incubation at 72°C. PCR products were run on 2% agarose gel and then stained with ethidium bromide (EtBr).

### 2.6. Measurement of Reactive Oxygen Species (ROS) Production

ROS level was measured using 2,7-dichlorofluorescein diacetate (DCFDA) fluorescence dye. Cells were incubated with 1 *μ*M DCFDA at 37°C for 30 min. Fluorescence intensity was measured by BD FACSCalibur flow cytometry (Becton Dickinson, Franklin Lakes, NJ).

### 2.7. Western Blotting

Cells (1 × 10^6^ cells/mL) were treated with various concentrations of melatonin (0, 1, or 2 mM) for 24 h, lyzed in lysis (50 mM Tris-HCl, pH 7.4, 150 mM NaCl, 1% Triton X-100, 0.1% SDS, 1 mM EDTA, 1 mM Na_3_VO_4_, 1 mM NaF, and 1x protease inhibitor cocktail) on ice, and spun down at 14,000 ×g for 20 min at 4°C. The supernatants were collected and quantified for protein concentration by using RC DC protein assay kits (Bio-Rad, Hercules, CA, USA). The protein samples were separated on 4–12% NuPAGE Bis-Tris gels (Novex, Carlsbad, CA, USA) and transferred to a Hybond ECL transfer membrane for detection with antibodies for Cx26, Cx30, Cx43 and phosphor-Cx43 (Santa Cruz Biotechnologies, Santa Cruz, CA, USA), phospho-JNK, JNK, phospho-p38 MAPK, p38 MAPK, phospho-ERK, and ERK (Cell signaling Technology, Beverly, MA, USA), and *β*-actin (Sigma, St. Louis, MO, USA).

### 2.8. Statistical Analyses

All data were expressed as means ± SD. The statistically significant differences between control and melatonin-treated groups were calculated by ANOVA test followed by a post hoc analysis (Tukey or Dunnett's multiple-comparison test) using Prism software 5 (GraphPad Software, Inc., San Diego, CA, USA).

## 3. Results

### 3.1. Melatonin and H_2_O_2_ Exerted Weak Cytotoxicity in HaCaT Cells

**Figure 1:**
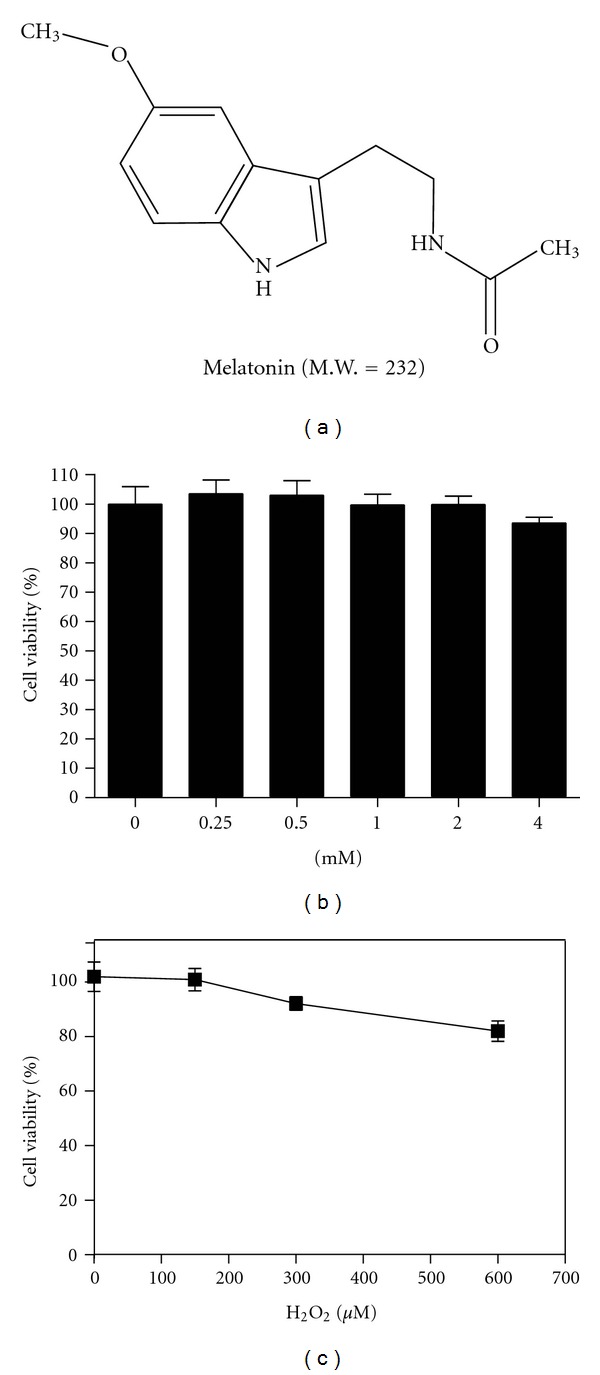
Chemical structure and cytotoxicity of melatonin. (a) Chemical structure of melatonin. Cytotoxicity of melatonin (b) and H_2_O_2_ (c) in HaCaT cells. Cytotoxicity of melatonin and H_2_O_2_ was evaluated in HaCaT cells by MTT assay. Cells were plated onto 96-well microplates (1 × 10^4^ cells/well) and treated with various concentrations of melatonin (0, 0.25, 0.5, 1, 2, or 4 mM) and H_2_O_2_ (0, 150, 300, or 600 *μ*M) for 24 h. Data were expressed as means ± SD of three independent experiments.

To determine nontoxic concentrations of melatonin and H_2_O_2_, the cytotoxic effects of melatonin and H_2_O_2_ were evaluated in HaCaT cells by MTT assay. Cells were exposed to various concentrations of melatonin (0, 0.25, 0.5, 1, 2, or 4 mM) and H_2_O_2_ (0, 150, 300, or 600 *μ*M) for 24 h, and then MTT assay was performed. As shown in Figures 1(b) and 1(c), melatonin and H_2_O_2_ showed weak cytotoxic effect in HaCaT cells. Thus, a concentration of 300 *μ*M H_2_O_2_ was used for all experiments.

### 3.2. Melatonin Reduced ROS Production and Facilitated the Decreased GJIC Activity in H_2_O_2_-Treated HaCaT Cells

**Figure 2:**
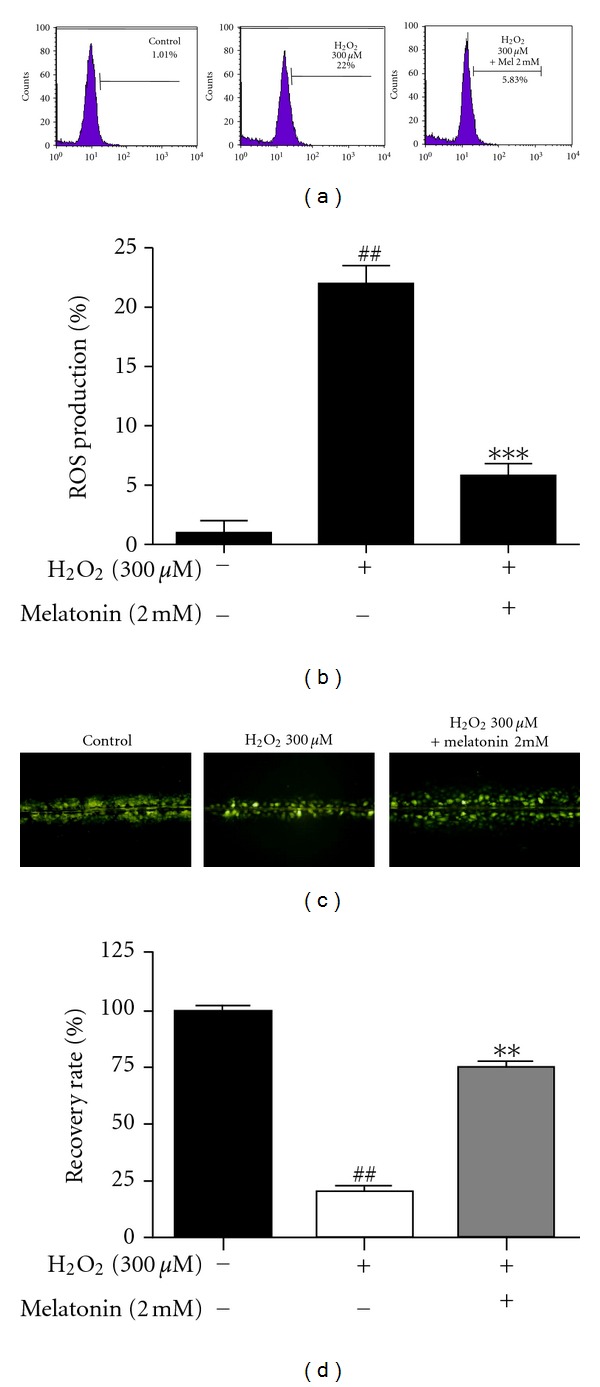
Melatonin reduced ROS production and facilitated the decreased GJIC activity in H_2_O_2_-treated HaCaT cells. (a) Cells were exposed to H_2_O_2_ (300 *μ*M) with or without melatonin (2 mM) for 24 h. ROS generation (%) was measured using ROS-sensitive fluorometric probe 2,7-dichlorofluorescein diacetate (DCFDA) by flow cytometric analysis. (b) Quantified graph for ROS production. Data represent means ± SD. ^##^ *P* < 0.01 versus untreated control. ***P < 0.001 versus melatonin treated cells. (c) GJIC was assessed using the scrape-loading/dye-transfer (SL/DT) method under an inverted fluorescence microscope (100x). (d) Quantification of recovery rate.

H_2_O_2_ is well known to produce free radicals to inhibit gap junctional intercellular communication [26]. As shown in Figure 2(a), melatonin reduced ROS production to 5.83% compared to H_2_O_2_-treated control (22%) in HaCaT cells. Consistently, melatonin enhanced intercellular communication disturbed by H_2_O_2_ in HaCaT cells by scrape-loading dye-transfer assay as shown in Figures 2(c) and 2(d).

### 3.3. Melatonin Significantly Enhanced the Expression of Cx26 and Cx43 at mRNA and Protein Levels, but Not That of Cx30 in H_2_O_2_-Treated HaCaT Cells

**Figure 3:**
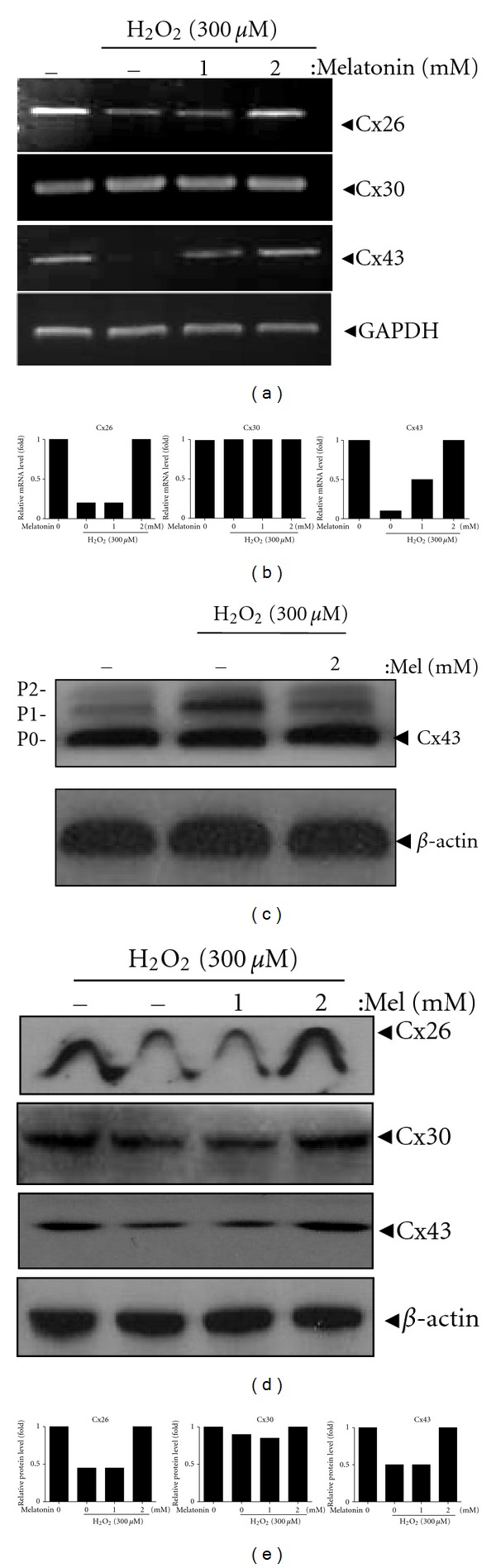
Melatonin significantly enhanced the expression of Cx26 and Cx43 at mRNA and protein levels, but not that of Cx30 in H_2_O_2_-treated HaCaT cells. (a) Cells were exposed to H_2_O_2_ (300 *μ*M) with or without melatonin (1 or 2 mM) for 24 h. (a) mRNAs expressions of Cx26, Cx30, and Cx43 were analyzed by RT-PCR. Grapes represent relative level of Cx26, Cx30, and Cx43/GAPDH. (b) Quantification of mRNAs expression. Phosphorylation of Cx43 (c) and protein expressions of Cx26, Cx30, and Cx43 (d) in melatonin-H_2_O_2_-treated cells were analyzed by western blot. (e) Grapes represent relative level of Cx26, Cx30, and Cx43/*β*-actin.

The phosphorylation of the gap junction protein Cx43 is directly associated to functional GJIC [27]. To investigate the effect of melatonin on connexins at mRNA and protein levels in H_2_O_2_-treated HaCaT cells, RT-PCR and western blot analyses were carried out. As shown in Figures 3(a) and 3(b), mRNA levels of Cx26 and Cx43 were reduced by H_2_O_2_-alone treatment, while melatonin enhanced the mRNA level of them in H_2_O_2_-treated HaCaT cells. mRNA level of Cx30 did not change in H_2_O_2_- or melatonin-treated cells. Consistently, melatonin increased the protein level of Cx26 and Cx43 in H_2_O_2_-treated HaCaT cells (Figures 3(d) and 3(e)). We also observed that melatonin suppressed the phosphorylation of Cx43 in H_2_O_2_-treated HaCaT cells (Figure 3(c)).

### 3.4. Melatonin Significantly Decreased the Phosphorylation of ERK Alone, but Not p38 MAPK or JNK in H_2_O_2_-Treated HaCaT Cells

**Figure 4:**
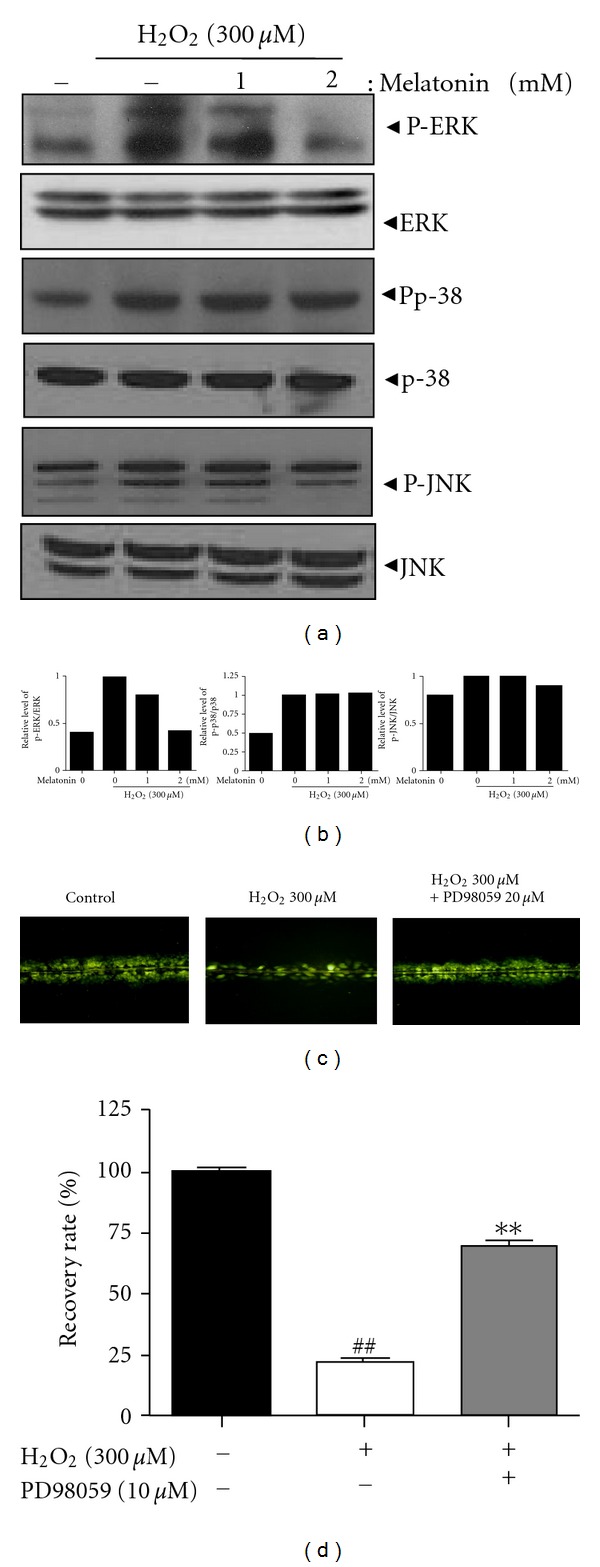
Melatonin significantly decreased the phosphorylation of ERK alone, but not p38 MAPK or JNK in H_2_O_2_-treated HaCaT cells. Cells were exposed to H_2_O_2_ (300 *μ*M) with or without melatonin (1 or 2 mM) for 24 h. (a) Western blotting was performed for phospho-ERK, ERK, phospho-p38, p38, phospho-JNK, and JNK. (b) Graphs represent relative level of phospho-ERK/ERK, phospho-p38/p38, and phospho-JNK/JNK. (c) Effect of ERK inhibitor PD98059 on GJIC using the SL/DT method. (d) Quantification of recovery rate.

The effect of melatonin on MAPK signaling was investigated in H_2_O_2_-treated HaCaT cells. Melatonin attenuated the phosphorylation of ERK, but did not significantly affect that of p38 MAPK and JNK in H_2_O_2_-treated HaCaT cells, while H_2_O_2_ activated the phosphorylation of ERK, p38, and JNK proteins as shown in Figures 4(a) and 4(b). Next, in order to confirm that the GJIC by H_2_O_2_ is mediated by ERK pathway, we used the ERK inhibitor PD98059. As shown in Figures 4(c) and 4(d), ERK inhibitor PD98059 effectively recovered the decreased activity of GJIC in H_2_O_2_-treated HaCaT cells.

### 3.5. Combined Treatment of Melatonin and Vitamin C at Low Concentrations Exerted the Synergy in Reducing ROS Production in H_2_O_2_-Treated HaCaT Cells

**Figure 5:**
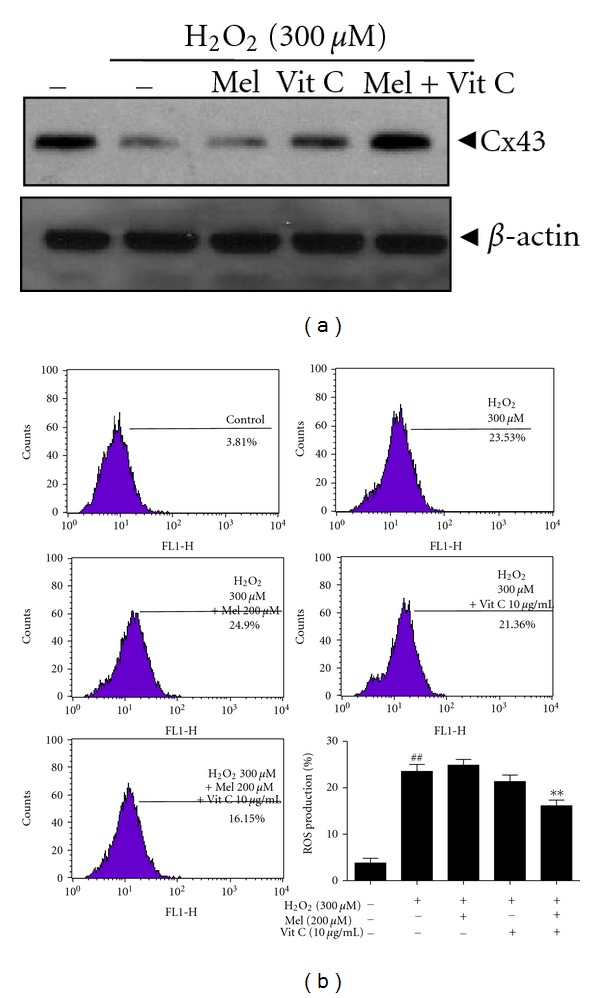
Combined treatment of melatonin and vitamin C at low concentrations exerted the synergy in reducing ROS production in H_2_O_2_-treated HaCaT cells. H_2_O_2_-treated HaCaT cells were exposed in the absence or presence of melatonin (200 *μ*M), vitamin C (10 *μ*g/mL), and melatonin plus vitamin C for 24 h. (a) Western blotting was performed for Cx43 and *β*-actin. (b) ROS generation (%) was measured using ROS-sensitive fluorometric probe 2,7-dichlorofluorescein diacetate (DCFDA) by flow cytometric analysis. Graph represents quantification for ROS production.

In order to evaluate the synergistic effect of melatonin with other antioxidant, we used vitamin C. As shown in Figure 5(a), melatonin (200 *μ*M) or vitamin C (10 *μ*g/mL) alone at low concentration did not affect Cx34 in H_2_O_2_-treated HaCaT cells. In contrast, combined treatment of melatonin and vitamin C promoted the expression of Cx34. Similarly, though melatonin at 2 mM suppressed ROS generation induced by H_2_O_2_, low concentration (200 *μ*M) of melatonin did not affect ROS production as in Figure 5(b). As shown in Figure 5(b), melatonin (200 *μ*M) or vitamin C (10 *μ*g) alone did not affect ROS production, but combination of melatonin and vitamin C significantly reduced ROS production to 16.15% compared to H_2_O_2_-treated control (23.56%).

## 4. Discussion

H_2_O_2_ plays an important role in the multistep process of carcinogenesis and directly promotes transformation in many *in vivo* and *in vitro* model systems [28–30]. In the present study, melatonin suppressed ROS production and facilitated H_2_O_2_-mediated inhibition of GJIC in HaCaT cells, implying the antioxidant and anti-carcinogenic potential of melatonin, which was supported by previous studies that the carcinogenicity of H_2_O_2_ is attributable to the inhibition of GJIC [31]. Likewise, antioxidants such as vitamin C and quercetin protect against the disruption of GJIC induced by H_2_O_2_ [32].

There are several lines of evidences that malignant lesions reveal abnormal expression of connexins and decreased GJIC [33–35]. The function of GJIC can be modulated at the multi-stages during the turnover of connexins by transcriptional, translational, and posttranscriptional mechanisms. Hence, prevention or inhibition of decreased GJIC can be an important target for cancer therapy. As suggested, H_2_O_2_ induced downregulation of connexins, thereby disrupting the GJIC system [5]. Here we found that melatonin recovered the reduced phosphorylation of Cx26 and Cx43 induced by H_2_O_2_ at protein and mRNA levels, but not that of Cx30 in H_2_O_2_-treated HaCaT cells, indicating that melatonin regulates GJIC via activation of Cx26 and Cx43 signaling.

MAPKs are considered to play important roles in GJIC [36]. Also, ROS-activated MAPK cascades phosphorylate the various proteins involved in cell growth and development [37]. Previous studies revealed that H_2_O_2_-dependent ERK and p38 kinase activation lead to depressed GJIC and enhanced connexin degradation [36]. However, in the current study, melatonin significantly decreased the phosphorylation of ERK alone, but not p38 MAPK or JNK. Furthermore, ERK inhibitor PD98059 effectively recovered the lowered activity of GJIC in H_2_O_2_-treated HaCaT cells, suggesting the critical role of ERK in recovering the decreased GJIC activity by H_2_O_2_. Interestingly, combined treatment of melatonin (200 *μ*M) and vitamin C (10 *μ*g/mL) that do not affect ROS production significantly reduced ROS production in H_2_O_2_-treated HaCaT cells, implying the synergistic effect of melatonin and vitamin C at low concentrations. However, it is also required to confirm this synergistic effect in small animals or humans in the near future.

**Figure 6:**
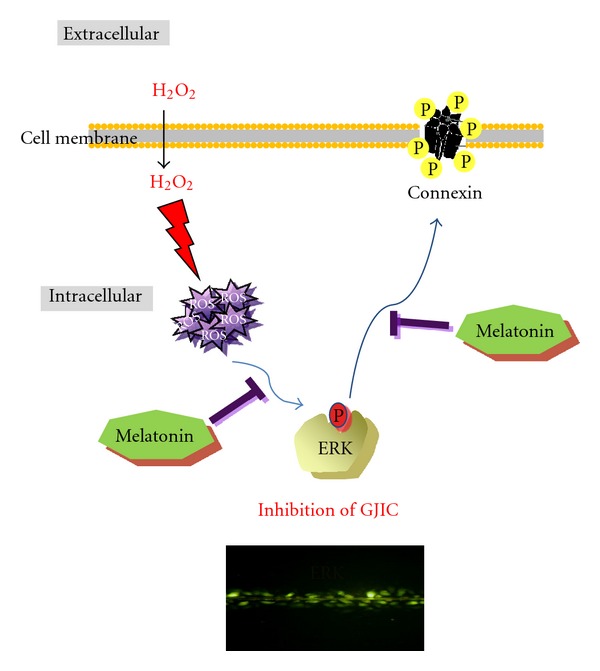
Molecular mechanism of melatonin facilitated GJIC in H_2_O_2_-treated HaCaT cells.

In summary, melatonin showed weak cytotoxicity in HaCaT cells, reduced ROS production, recovered the disturbed GJIC, enhanced the expression of Cx26 and Cx43 at mRNA and protein levels, suppressed the phosphorylation of ERK, and enhanced synergy with vitamin C in H_2_O_2_-treated HaCaT cells (Figure 6). Overall, our findings suggest that melatonin recovers decreased GJIC via enhancement of Cx26 and Cx43 and inhibition of ROS production and ERK phosphorylation.

## References

[1] Lowenstein WR (1979). Junctional intercellular communication and the control of growth. *Biochimica et Biophysica Acta*.

[2] Zampighi G (1980). On the structure of isolated junctions between communicating cells. *In Vitro*.

[3] Lee KM, Kwon JY, Lee KW, Lee HJ (2009). Ascorbic acid 6-palmitate suppresses gap-junctional intercellular communication through phosphorylation of connexin 43 via activation of the MEK-ERK pathway. *Mutation Research*.

[4] Salomon D, Masgrau E, Vischer S (1994). Topography in mammalian connexins in human skin. *Journal of Investigative Dermatology*.

[5] Trosko JE, Ruch RJ (1998). Cell-cell communication in carcinogenesis. *Frontiers in Bioscience*.

[6] Lampe PD, Lau AF (2004). The effects of connexin phosphorylation on gap junctional communication. *International Journal of Biochemistry and Cell Biology*.

[7] Lau AF, Kurata WE, Kanemitsu MY (1996). Regulation of connexin43 function by activated tyrosine protein kinases. *Journal of Bioenergetics and Biomembranes*.

[8] Cho JH, Cho SD, Hu H (2002). The roles of ERK1/2 and p38 MAP kinases in the preventive mechanisms of mushroom *Phellinus linteus* against the inhibition of gap junctional intercellular communication by hydrogen peroxide. *Carcinogenesis*.

[9] Upham BL, Gužvić M, Scott J (2007). Inhibition of gap junctional intercellular communication and activation of mitogen-activated protein kinase by tumor-promoting organic peroxides and protection by resveratrol. *Nutrition and Cancer*.

[10] Huang RP, Peng A, Golard A (2001). Hydrogen peroxide promotes transformation of rat liver non-neoplastic epithelial cells through activation of epidermal growth factor receptor. *Molecular Carcinogenesis*.

[11] Reiter RJ, Tan DX, Fuentes-Broto L (2010). Melatonin: a multitasking molecule. *Progress in Brain Research*.

[12] Stehle JH, Saade A, Rawashdeh O (2011). A survey of molecular details in the human pineal gland in the light of phylogeny, structure, function and chronobiological diseases. *Journal of Pineal Research*.

[13] Bonnefont-Rousselot D, Collin F, Jore D, Gardès-Albert M (2011). Reaction mechanism of melatonin oxidation by reactive oxygen species *in vitro*. *Journal of Pineal Research*.

[14] Galano A, Tan DX, Reiter RJ (2011). Melatonin as a natural ally against oxidative stress: a physicochemical examination. *Journal of Pineal Research*.

[15] Wu UI, Mai FD, Sheu JN (2011). Melatonin inhibits microglial activation, reduces pro-inflammatory cytokine levels, and rescues hippocampal neurons of adult rats with acute *Klebsiella pneumoniae* meningitis. *Journal of Pineal Research*.

[16] Belyaev O, Herzog T, Munding J (2011). Protective role of endogenous melatonin in the early course of human acute pancreatitis. *Journal of Pineal Research*.

[17] Raghavendra V, Kaur G, Kulkarni SK (2000). Anti-depressant action of melatonin in chronic forced swimming-induced behavioral despair in mice, role of peripheral benzodiazepine receptor modulation. *European Neuropsychopharmacology*.

[18] Lee SE, Kim SJ, Youn JP, Hwang SY, Park CS, Park YS (2011). MicroRNA and gene expression analysis of melatonin-exposed human breast cancer cell lines indicating involvement of the anticancer effect. *Journal of Pineal Research*.

[19] Koh W, Jeong SJ, Lee HJ (2011). Melatonin promotes puromycin-induced apoptosis with activation of caspase-3 and 5′-adenosine monophosphate-activated kinase-alpha in human leukemia HL-60 cells. *Journal of Pineal Research*.

[20] Jung-Hynes B, Schmit TL, Reagan-Shaw SR, Siddiqui IA, Mukhtar H, Ahmad N (2011). Melatonin, a novel Sirt1 inhibitor, imparts antiproliferative effects against prostate cancer *in vitro* in culture and in vivo in TRAMP model. *Journal of Pineal Research*.

[21] McGonnell IM, Green CR, Tickle C, Becker DL (2001). Connexin43 gap junction protein plays an essential role in morphogenesis of the embryonic chick face. *Developmental Dynamics*.

[22] Vinken M, Henkens T, De Rop E, Fraczek J, Vanhaecke T, Rogiers V (2008). Biology and pathobiology of gap junctional channels in hepatocytes. *Hepatology*.

[23] Zhou Y, Mi MT, Zhu JD, Zhang QY (2003). Effects of lovastatin on proliferation and gap junctional intercellular communication of human breast cancer cell MCF-7. *Ai Zheng*.

[24] Gakhar G, Schrempp D, Nguyen TA (2009). Regulation of gap junctional intercellular communication by TCDD in HMEC and MCF-7 breast cancer cells. *Toxicology and Applied Pharmacology*.

[25] El-Fouly MH, Trosko JE, Chang CC (1987). Scrape-loading and dye transfer. A rapid and simple technique to study gap junctional intercellular communication. *Experimental Cell Research*.

[26] Upham BL, Kang KS, Cho HY, Trosko JE (1997). Hydrogen peroxide inhibits gap junctional intercellular communication in glutathione sufficient but not glutathione deficient cells. *Carcinogenesis*.

[27] Wilson MR, Close TW, Trosko JE (2000). Cell population dynamics (apoptosis, mitosis, and cell-cell communication) during disruption of homeostasis. *Experimental Cell Research*.

[28] Okamoto M, Oyasu R (1997). Transformation *in vitro* of a nontumorigenic rat urothelial cell line by tumor necrosis factor-*α*. *Laboratory Investigation*.

[29] Ruch RJ, Cheng SJ, Klaunig JE (1989). Prevention of cytotoxicity and inhibition of intercellular communication by antioxidant catechins isolated from Chinese green tea. *Carcinogenesis*.

[30] Muehlematter D, Larsson R, Cerutti P (1988). Active oxygen induced DNA strand breakage and poly ADP-ribosylation in promotable and non-promotable JB6 mouse epidermal cells. *Carcinogenesis*.

[31] Huang RP, Peng A, Hossain MZ, Fan Y, Jagdale A, Boynton AL (1999). Tumor promotion by hydrogen peroxide in rat liver epithelial cells. *Carcinogenesis*.

[32] Lee KW, Lee HJ, Kang KS, Lee CY (2002). Preventive effects of vitamin C on carcinogenesis. *The Lancet*.

[33] Sulkowski S, Sulkowska M, Skrzydlewska E (1999). Gap junctional intercellular communication and carcinogenesis. *Polish Journal of Pathology*.

[34] Temme A, Buchmann A, Gabriel HD, Nelles E, Schwarz M, Willecke K (1997). High incidence of spontaneous and chemically induced liver tumors in mice deficient for connexin32. *Current Biology*.

[35] Kamibayashi Y, Oyamada Y, Mori M, Oyamada M (1995). Aberrant expression of gap junction proteins (connexins) is associated with tumor progression during multistage mouse skin carcinogenesis *in vivo*. *Carcinogenesis*.

[36] Hwang JW, Park JS, Jo EH (2005). Chinese cabbage extracts and sulforaphane can protect H_2_O_2_-induced inhibition of gap junctional intercellular communication through the inactivation of ERK1/2 and p38 MAP kinases. *Journal of Agricultural and Food Chemistry*.

[37] Kuruganti PA, Wurster RD, Lucchesi PA (2002). Mitogen activated protein kinase activation and oxidant signaling in astrocytoma cells. *Journal of Neuro-Oncology*.

